# To use or not to use dipyrone?

**DOI:** 10.1590/S1516-31802002000200009

**Published:** 2002-03-02

**Authors:** Marcelo Eduardo Bigal

I read with interest the excellent editorial by Benseñor,^1^ discussing the question about whether to use dipyrone or not. Dipyrone is a pyrazolone-derived, non-opiate analgesic drug. Its effectiveness has been shown in various painful situations, including headaches. Several studies have failed to show any association between dipyrone use and aplastic anemia, or have shown a very low risk. A study comparing the risk of serious adverse effects after the use of dipyrone, aspirin, diclofenac and paracetamol for short periods of time showed that the excess mortality attributed to each of these drugs was as follows: dipyrone — 25/100 million, acetyl salicylic acid – 185/100 million, diclofenac — 592/100 million, paracetamol – 20/100 million. The authors concluded that the risk of agranulocytosis secondary to dipyrone would have to be 300 times higher for the excess mortality attributed to this drug to be comparable to that of diclofenac.^2^

So, if the drug is safe and with a good efficacy profile, do we still have a question? We have just finished two randomized, placebo-controlled and double blind studies evaluating the efficacy of dipyrone in patients that sought emergency room assistance with the complaint of acute migraine^3^ or acute episodic tension-type headache.^4^ We made calculations of the therapeutic gain (TG, defined as active response minus placebo response) and the number needed to treat (NNT, the reciprocal of the TG, which indicates the number of patients that must receive the active drug for at least one of them to have a true benefit). We found the following results, one hour after the administration of the substances: 1) Migraine without aura: TG = 49.2%; NNT = 2.03; 2) migraine with aura: TG = 44.0%; NNT = 2.3; 3) episodic tension-type headache: TG = 60.0%; NNT = 1.6. Just for comparison purposes, parenteral diclofenac, a recognized option for treating acute headaches, had TG ranging from 10.0% (episodic tension-type headache) to 36.7% (migraine with aura). The efficacy of parenteral dipyrone was at least as good as the better triptans available, which are specific anti-migraine options that are very good and safe in the treatment of acute migraines.

We think that the major advantage of triptans in relation to parenteral dipyrone is the avoidance of seeking out emergency room assistance to receive parenteral drugs, making it easier for patients to quickly return to their usual activities and decreasing the indirect costs of the disease. It is our opinion that this class of drugs is the first choice in acute outpatient treatment for migraines. But we also believe that dipyrone is at least comparable with the most modern and first-line treatments, and is an excellent choice for use in emergency rooms.

Benseñor concluded the editorial with the following words: "In the light of evidence-based medicine and forgetting E.R. episodes: it is time to take decisions based on evidence and not on prejudices". It is my belief that the evidence strongly supports the use of dipyrone. It is surely an excellent option for treating very common painful diseases like acute headaches in the emergency room setting.
